# Genic and non-genic SNP contributions to additive and dominance genetic effects in purebred and crossbred pig traits

**DOI:** 10.1038/s41598-022-07767-3

**Published:** 2022-03-09

**Authors:** Mahshid Mohammadpanah, Ahmad Ayatollahi Mehrgardi, Hélène Gilbert, Catherine Larzul, Marie-José Mercat, Ali Esmailizadeh, Mehdi Momen, Llibertat Tusell

**Affiliations:** 1 Department of Animal Science, Faculty of Agriculture, Shahid Bahonar University of Kerman (SBUK), Kerman, Iran; 2 GenPhySE, Université de Toulouse, INRAE, 31326 Castanet-Tolosan, France; 3 IFIP/Alliance R&D, La Motte au Vicomte, 35651 Le Rheu, France; 4 Department of Surgical Sciences, School of Veterinary Medicine, University of Wisconsin-Madison, Madison, WI 53706 USA; 5 Animal Breeding and Genetics Program, Institute of Agriculture and Food Research and Technology (IRTA), Torre Marimon s/n, Caldes de Montbui, 08140 Barcelona, Spain

**Keywords:** Biotechnology, Genetics

## Abstract

The present research has estimated the additive and dominance genetic variances of genic and intergenic segments for average daily gain (ADG), backfat thickness (BFT) and pH of the semimembranosus dorsi muscle (PHS). Further, the predictive performance using additive and additive dominance models in a purebred Piétrain (PB) and a crossbred (Piétrain × Large White, CB) pig population was assessed. All genomic regions contributed equally to the additive and dominance genetic variations and lead to the same predictive ability that did not improve with the inclusion of dominance genetic effect and inbreeding in the models. Using all SNPs available, additive genotypic correlations between PB and CB performances for the three traits were high and positive (> 0.83) and dominance genotypic correlation was very inaccurate. Estimates of dominance genotypic correlations between all pairs of traits in both populations were imprecise but positive for ADG-BFT in CB and BFT-PHS in PB and CB with a high probability (> 0.98). Additive and dominance genotypic correlations between BFT and PHS were of different sign in both populations, which could indicate that genes contributing to the additive genetic progress in both traits would have an antagonistic effect when used for exploiting dominance effects in planned matings.

## Introduction

Crossbreeding schemes have largely driven genetic progress in pigs during the last 40–50 years^1^. With crossbreeding, specific alleles of parental purebreds are inherited by the offspring generating many heterozygous loci contributing to increase the favorable heterosis effects on the traits of interest. Dominant gene action has a major role on heterosis^2,3^. Predominantly, genetic selection in pigs is performed on purebred lines using purebred data and accounting solely for additive genetic effects, whereas crossbred performance is the ultimate product to improve as a correlated response^4^. If the genetic correlation between purebred and crossbred performances substantially differs from unity, a combined crossbred and purebred selection method is advised^5^. In addition to selection strategies, if dominance effects are important, assortative mating strategies might enhance the total genetic values of the crossbred offspring at a given generation^6–10^. Thus, estimates of additive and dominance genetic parameters for traits of interest and for purebred and crossbred performances are required to determine the best breeding and management strategies in crossbreeding schemes. With technological developments in the recent years, these strategies can now be based on marker information and several genomic models to account for dominance effects are available for purebred and crossbred genetic evaluations (e.g.^9–15^).

In addition, separately estimating and accounting for additive and dominance effects of genic and non-genic regions could provide a better understanding of the genetic architecture and potentially improve predictive ability of models for complex traits in pigs. The contribution of genic and non-genic regions of the genome to additive genetic variance has been investigated in humans and some livestock species^16–18^ to provide a better understanding of the genetic architecture and improve predictive ability of models for complex traits. A further step is to investigate the contribution of these genome regions to dominance genetic variance across purebred and crossbred populations.

The present research has estimated the additive and dominance genetic variances due to genic and non-genic segments for average daily gain (ADG), backfat thickness (BF) and pH of the semimembranosus dorsi muscle (PHS) and their predictive performance in a purebred Piétrain (PB) and a crossbred (Piétrain × Large White, CB) pig population. Estimates of additive and dominance genotypic correlations between purebred and crossbred performances for these traits as well as between traits within PB and CB populations are also provided.

## Material and methods

### Ethics approval

Data used for this study was originated in the UtOpIGe project ANR-10-GENOM_BTV-015. All experimental protocols of the project were approved on the 01/23/2013 (R-2012-NM-01) by the local ethic committee (Comité Rennais d’Ethique en matière d’Expérimentation Animale). The Comité Rennais d’Ethique en matière d’Expérimentation Animale is registered in the French National Committee of ethical reflexion on animal experimentation number 7 (Comité National de Refléxion Ethique sur l’Expérimentation Animale). All methods were carried out in accordance with guidelines and the French regulation in Animal research (articles R214-87 to R214-137 of the French rural code https://www.recherche-animale.org/sites/default/files/c_rural_2013.pdf) updated by the 2013–118 decree and five orders from February 1st 2013, published on February 7th, according to the 2010/63 directive from the EU. This regulation is under the responsibility of the French Ministry of Agriculture.

The study was carried out in compliance with the ARRIVE guidelines (http://www.nc3rs.org.uk/page.asp?id=1357).

### Animal material

Animals were provided by the three French breeding companies of the Alliance R&D association (composed of Axiom, Choice Genetics France, Nucléus and IFIP) involved in the UtOpIGe project ANR-10-GENOM_BTV-015. We used 636 purebred Piétrain (PB) and 720 crossbred Piétrain × Large White (CB) entire “intact” male piglets produced on selection and multiplication farms and tested at a single test station INRA UE3P France Génétique Porc phenotyping station (UE3P, INRA, 2018. Unité expérimentale Physiologie ET Phénotypage des Porcs, France, https://doi.org/10.15454/1.5573932732039927E12). Both, PB and CB animals were descendants of 90 Piétrain boars. They entered the test station facilities in Le Rheu (France) at approximately 5 weeks of age and were slaughtered at a fixed weight of 110 kg (at 5–6 months of age).

### Phenotypes

Animals were misspelled weighed at the beginning (when the animals reached approximately 35 kg) and at the end of the test period (when they reached 110 kg). Average daily gain (ADG, kg/day) was calculated as the body weight gained during the test period divided by the duration of the period. Rib back fat thickness (BFT, mm) was measured on carcass with the Capteur Gras Maigre method^19^. At the slaughterhouse, carcasses were chilled in a cooling room at 4 °C for 24 h. Ultimate pH of the semimembranosus dorsi muscle (PHS, pH units) was measured using a Xerolyt electrode (Mettler-Toledo, Australia) and a Sydel pH meter (Sydel, France) at 24 h post- mortem. Further details regarding complete data collection and experimental design can be found in Tusell et al.^10^.

### Genotypes

Animals were genotyped using the Illumina Porcine SNP60 Bead Chip (Illumina, Inc., San Diego). Single nucleotide polymorphisms (SNPs) with a call rate lower than 0.90 and a minor allele frequency lower than 0.05 were removed. For the remaining SNPs. Missing genotypes were imputed using a naïve method that sampled genotypes with probability weights of the allele frequencies at each locus. The missing genotypes were then replaced with these sampled genotypes. Animals that presented Mendelian inconsistencies with their parents were discarded. After quality control, 46,816 SNPs were included in the analyses. Due to separate data edition, the number of animals with records and number of SNPs retained for the analyses slightly differed for each trait. Summary statistics of the three analyzed phenotypes in the two populations are presented in Table 1.

**Table 1:** Summary statistics of the purebred (PB) and crossbred (CB) phenotype data.

Trait	PB		CB	
	Number of records	Mean (SD)	Number of records	Mean (SD)
ADG	636	940 (94)	720	1003 (92)
BFT	607	10.14 (1.81)	620	11.51 (2.13)
PHS	632	5.68 (0.18)	727	5.72 (0.19)

*ADG* average daily gain (g/day), *BFT* backfat thickness (mm), *PHS* pH of the semimembranosus muscle (pH units).

### SNP annotation

Chromosome information of SNPs (i.e. the map file containing SNP-ID and RS-Numbers) was downloaded from the Animal Genome Database (https://www.animalgenome.org/repository/pig/). The SNPs that did not have RS number or a non-unique RS number were discarded leading to 39,727 SNPs available for the analysis. The physical positions of the SNPs obtained through Ensembl database (https://useast.ensembl.org/Sus_scrofa/Tools/VEP) for pig (*Sus Scrofa,* Assembly: Sscrofa11.1, accession date: December 2018) allowed to locate each SNP into a genic or an intergenic region.

SNPs were classified in three categories. Genic region category (Genic, 19,672 SNPs) encompassed the SNPs annotated into the following categories: introns (15,824 SNPs), synonymous (321 SNPs), upstream the gene (1,595 SNPs), downstream the gene (1,338 SNPs), 5′ (77 SNPs) and 3′ untranslated regions (UTR) (382 SNPs), missense (108 SNPs), other exon mutations (27 SNPs). Intergenic region category (Intergenic, 20,055 SNPs) encompassed all SNPs annotated outside these genic regions. All region category (All) included the 39,727 SNPs.

### Statistical analysis

#### Genic and intergenic additive and dominance variances in purebred and crossbred populations

To explore the additive and dominance variance explained by the SNPs of the categories (i.e., Intergenic, Genic and All SNPs) in ADG, BF and PHS in the two populations, the following general univariate model was fitted separately for each trait, population and genomic region^9,14^:

$$\mathbf{y}={\mathbf{X}}_{1}{\varvec{\upbeta}}+\mathbf{f}b+{\mathbf{X}}_{2}\mathbf{p}+\mathbf{Z}\mathbf{a}+\mathbf{W}\mathbf{d}+\mathbf{e}$$

where $\mathbf{y}$ was the phenotypic value of individuals, ${\varvec{\upbeta}}$ is a vector of systematic effects and $\mathbf{p}$ a vector of pen nested within batch random effects. Term $\mathbf{e}$ is the vector of residual effects. Terms ${\mathbf{X}}_{1}$ and ${\mathbf{X}}_{2}$ are incidence matrices that assign systematic and nested within batch effects to the phenotypes, respectively. Model for ADG included the effects of weight at the beginning of test (covariate), and pen nested within batch effect (random effect, 65 levels). Model for BFT included the effects of hot carcass weight (covariate) and pen nested within batch effect (random effect, 62 levels). Model for PHS included the effects of hot carcass weight (covariate) and date of slaughter (systematic effect, 43 levels). Term $\mathbf{f}$ is a vector of inbreeding coefficients calculated as the average homozygosity per individual and $b$ is the inbreeding depression coefficient^14^. Terms $\mathbf{a}$ and $\mathbf{d}$ are the vectors of animal additive and dominance genotypic effects, respectively. Terms $\mathbf{Z}$ and $\mathbf{W}$ are incidence matrices relating additive and dominance genotypic effects to either PB or CB animals with −1, 0, 1 (additive) and 0, 1, 0 (dominance) values for the *AA*, *Aa* and *aa* genotypes, respectively. Additive and dominance genotypic (co)variances were modelled as $\mathbf{G}=\frac{\mathbf{Z}{\mathbf{Z}}^{\boldsymbol{^{\prime}}}}{\left\{tr([\mathbf{Z}{\mathbf{Z}}^{\boldsymbol{^{\prime}}}])/n\right\}}{\sigma }_{A*}^{2}$ and $\mathbf{D}=\frac{\mathbf{W}\mathbf{W}}{\left\{tr([\mathbf{W}{\mathbf{W}}^{\boldsymbol{^{\prime}}}])/n\right\}}{\sigma }_{D*}^{2}$^12^, where ${\sigma }_{A*}^{2}$ and ${\sigma }_{D*}^{2}$ are the estimated variance components, and $n$ the number of animals.

A Bayesian framework was adopted for inference. The prior distributions for the parameters of the model were $P({\varvec{\beta}},b)\sim k$, $P\left({\varvec{p}}|{\sigma }_{p}^{2} \right)\sim N(0, {\mathbf{I}\sigma }_{p}^{2})$, $P(\mathbf{a}|{\mathbf{G}, \sigma }_{A*}^{2})\sim N(0, \mathbf{G}{\sigma }_{A*}^{2})$, $P(\mathbf{d}|{\mathbf{D}, \sigma }_{D*}^{2})\sim N(0, \mathbf{D}{\sigma }_{D*}^{2})$ and $P\left({\varvec{e}}|{\sigma }_{e}^{2}\right)\sim N(0, {\mathbf{I}\sigma }_{e}^{2})$ where $k$ is a constant, $\mathbf{I}$ is an identity matrix, and ${\sigma }_{p}^{2}$ and ${\sigma }_{e}^{2}$ are the nested within batch and residual variances, respectively.

The variance components were estimated either using all the 39,727 available SNPs (All), the 19,672 SNPs located in the genic regions (Genic) or the 20,055 SNPs located in the intergenic regions (Intergenic). Thus, for each genomic region, the SNPs included in $\mathbf{Z}$ and $\mathbf{W}$ differed according to the genomic region used (i.e., All, Genic or intergenic SNPs).

Following^12^, the variance components ${\sigma }_{A\boldsymbol{*}}^{2}$ and ${\sigma }_{D*}^{2}$ estimated in the genotypic models were then used to retrieve the additive and dominance SNP variances that, together with the allelic frequencies of each population, allowed to obtain the additive and dominance deviation variances for the two populations across the three analysed traits. Hence, with the SNPs of the three regions, three different models (using either Genic, Intergenic or All SNPs) were implemented per trait (ADG, BFT and PHS) and per population (i.e. PB or CB) leading to 18 different models.

#### Predictive ability

Predictive ability of a model including only additive genetic effects and an inbreeding coefficient was compared to a model including additive and dominance effects and an inbreeding coefficient. These two models (additive model or additive and dominance model) were run separately for each combination of population (PB or CB), trait (ADG, BFT and PHS) and genomic region (Genic, Intergenic and All markers) leading to 36 different models. All models included the same systematic and non-genetic random effects described above for each of the three traits. Predictive ability of the models was evaluated by cross-validation (CV). Specifically, a four-fold CV scheme was used by attributing animals randomly to one of four separate subsets. From these four subsets, three folds were combined to create a training set and the remaining fold was used as testing set. Each of the four subsets was applied as a testing set only once. Because of the small size of the sample, the four-fold CV was replicated 10 times at random, and results were averaged over replications^20^. Predictive abilities were assessed via Pearson’s correlation between pre-adjusted phenotypes and predicted phenotypes in the testing sets.

#### Additive and dominance genotypic correlations between PB and CB populations

Additive and dominance genotypic correlations between PB and CB performances were estimated using all SNPs available and separately for each trait. In this case, PB and CB performances of the trait (i.e., ADG, BFT or PHS) were considered to be different traits in PB and CB populations and were jointly analyzed with a bivariate genotypic model accounting for additive and dominance effects^12^ and a genomic inbreeding coefficient^14^. The following general two-trait model was applied:

$$\left[\begin{array}{l}{\mathbf{y}}_{PB}\\ {\mathbf{y}}_{CB}\end{array}\right]=\left[\begin{array}{ll}{\mathbf{X}}_{1,PB}& 0\\ 0& {\mathbf{X}}_{1,CB}\end{array}\right]\left[\begin{array}{l}{{\varvec{\upbeta}}}_{PB}\\ {{\varvec{\upbeta}}}_{CB}\end{array}\right]+\left[\begin{array}{l}{\mathbf{f}}_{PB}\\ {\mathbf{f}}_{CB}\end{array}\right]\left[\begin{array}{l}{b}_{PB}\\ {b}_{CB}\end{array}\right]+\left[\begin{array}{ll}{\mathbf{X}}_{2,PB}& 0\\ 0& {\mathbf{X}}_{2,CB}\end{array}\right]\left[\begin{array}{l}{\mathbf{p}}_{PB}\\ {\mathbf{p}}_{CB}\end{array}\right]+\left[\begin{array}{ll}{\mathbf{Z}}_{A,PB}& 0\\ 0& {\mathbf{Z}}_{A,CB}\end{array}\right]\left[\begin{array}{l}{\mathbf{a}}_{PB}\\ {\mathbf{a}}_{CB}\end{array}\right]+\left[\begin{array}{ll}{\mathbf{Z}}_{D,PB}& 0\\ 0& {\mathbf{Z}}_{D,CB}\end{array}\right]\left[\begin{array}{l}{\mathbf{d}}_{PB}\\ {\mathbf{d}}_{CB}\end{array}\right]+\left[\begin{array}{l}{\mathbf{e}}_{PB}\\ {\mathbf{e}}_{CB}\end{array}\right]$$

Index $$k$$ (for $$k=PB, CB)$$ is used to denote either the PB ($$k=PB$$) or the CB ($$k=CB$$) populations. Term $${\mathbf{y}}_{k}$$ is a vector of phenotypes, $${{\varvec{\upbeta}}}_{k}$$ is a vector of systematic effects, $${\mathbf{p}}_{k}$$ is a vector of pen nested within batch effects (only included in ADG and BFT models), $${\mathbf{f}}_{k}$$ is a vector of inbreeding coefficients and its corresponding inbreeding depression coefficient ($${b}_{k}$$), and $${\mathbf{e}}_{k}$$ is a vector of residual effects. Terms $${\mathbf{X}}_{1,k}$$**, **$${\mathbf{X}}_{2,k}$$**,** $${\mathbf{Z}}_{A,k}$$ and $${\mathbf{Z}}_{D,k}$$ are incidence matrices that assign systematic, pen nested within batch effects, additive genotypic effects, and dominance genotypic effects to the phenotypes, respectively. There were no correlations assumed between pen nested within batch effects, between residual effects or between these effects and other random effects. The (co)variance matrix for the pen nested within batch effects is $$var\left[\begin{array}{l}{\mathbf{p}}_{PB}\\ {\mathbf{p}}_{CB}\end{array}\right]=\mathbf{I}\otimes \mathbf{P}=\mathbf{I}\otimes \left[\begin{array}{ll}{\sigma }_{p,PB}^{2}& 0\\ 0& {\sigma }_{p,CB}^{2}\end{array}\right]$$ and the (co)variance matrix for the residuals is $$var\left[\begin{array}{l}{\mathbf{e}}_{PB}\\ {\mathbf{e}}_{CB}\end{array}\right]=\mathbf{I}\otimes \mathbf{R}=\mathbf{I}\otimes \left[\begin{array}{ll}{\sigma }_{e,PB}^{2}& 0\\ 0& {\sigma }_{e,CB}^{2}\end{array}\right]$$, where $${\sigma }_{p,k}^{2} and {\sigma }_{e,k}^{2}$$ are the pen nested within batch and residual variances in the PB and CB populations, respectively. Vector $${\mathbf{a}}_{k}$$ is the vector of additive genetic effects and vector $${\mathbf{d}}_{k}$$ is the vector of dominance genotypic effects. The (co)variance matrix for the genotypic additive effects is:

$$var\left[\begin{array}{l}{\mathbf{a}}_{PB}\\ {\mathbf{a}}_{CB}\end{array}\right]$$= $${\mathbf{G}}_{0}\otimes \mathbf{G}=\left[\begin{array}{ll}{\sigma }_{A*PB}^{2}& {\sigma }_{A*PB,CB}\\ {\sigma }_{A*PB,CB}& {\sigma }_{A*CB}^{2}\end{array}\right]\otimes \frac{\mathbf{Z}{\mathbf{Z}}^{\mathbf{^{\prime}}}}{\left\{tr([\mathbf{Z}{\mathbf{Z}}^{\mathbf{^{\prime}}}])/n\right\}}$$where ${\sigma }_{A*k}^{2}$ is the additive genotypic variance in either PB or CB, and ${\sigma }_{A*PB,CB}$ is the additive genotypic covariance between PB and CB populations. Similarly, the (co)variance matrix for the genotypic dominance effects is:

$$var\left[\begin{array}{l}{\mathbf{d}}_{PB}\\ {\mathbf{d}}_{CB}\end{array}\right]={\mathbf{D}}_{0}\otimes \mathbf{D}=\left[\begin{array}{ll}{\sigma }_{D*PB}^{2}& {\sigma }_{D*PB,CB}\\ {\sigma }_{D*PB,CB}& {\sigma }_{D*CB}^{2}\end{array}\right]\otimes \frac{\mathbf{W}{\mathbf{W}}^{\mathbf{^{\prime}}}}{\left\{tr([\mathbf{W}{\mathbf{W}}^{\mathbf{^{\prime}}}])/n\right\}}$$

where ${\sigma }_{D*k}^{2}$ and ${\sigma }_{D*PB,CB}$ are the dominance genotypic variances in PB and CB, and the additive genotypic covariance between PB and CB populations. Terms $\mathbf{Z}$ and $\mathbf{W}$ are incidence matrices relating additive and dominance genotypic effects to the PB and CB animals coded as described in the models above. In this bivariate genotypic model, the $\mathbf{Z}$ and $\mathbf{W}$ matrices include all PB and CB animals and the model estimates additive and dominance genotypic covariances which cannot be interpreted in the same way as the statistical covariance of breeding values and dominance deviations^12^. The prior distributions for the parameters of the model were $P({\varvec{\beta}},b)\sim k$, $P\left({\varvec{p}}\left|\mathbf{P}\right.\right)\sim N(0, \mathbf{I}\otimes \mathbf{P})$, $P(\mathbf{a}\left|{\mathbf{G}}_{0}\right.)\sim {\varvec{M}}{\varvec{V}}N(0, {\mathbf{G}}_{0}\otimes \mathbf{G})$, $P(\mathbf{d}\left|{\mathbf{D}}_{0}\right.)\sim {\varvec{M}}{\varvec{V}}N(0, {\mathbf{D}}_{0}\otimes \mathbf{D})$ and $P\left({\varvec{e}}\left|\mathbf{R}\right.\right)\sim {\varvec{M}}{\varvec{V}}N(0, \mathbf{I}\otimes \mathbf{R})$.

#### Additive and dominance genotypic correlations between BFT, ADG and PHS in PB and CB populations

A tri-trait genotypic model including additive and dominance effects and a genomic inbreeding coefficient was used to estimate additive and dominance genotypic correlations between BFT, ADG and PHS within each PB and CB population. This approach allowed estimating (co)variances of additive and dominance genotypic effects between these three traits within purebred and crossbred populations. To achieve that, the following tri-trait model was applied separately in each population:

$$ \begin{aligned}\left[\begin{array}{l}{\mathbf{y}}_{ADG}\\ {\mathbf{y}}_{BFT}\\ {\mathbf{y}}_{PHS}\end{array}\right]&=\left[\begin{array}{lll}{\mathbf{X}}_{1,ADG}& 0& 0\\ 0& {\mathbf{X}}_{1,BFT}& 0\\ 0& 0& {\mathbf{X}}_{1,PHS}\end{array}\right]\left[\begin{array}{l}{{\varvec{\upbeta}}}_{ADG}\\ {{\varvec{\upbeta}}}_{BFT}\\ {{\varvec{\upbeta}}}_{PHS}\end{array}\right]+\left[\begin{array}{l}{\mathbf{f}}_{ADG}\\ {\mathbf{f}}_{BFT}\\ {\mathbf{f}}_{PHS}\end{array}\right]\left[\begin{array}{l}{b}_{ADG}\\ {b}_{BFT}\\ {b}_{PHS}\end{array}\right]+\left[\begin{array}{lll}{\mathbf{X}}_{2,ADG}& 0& 0\\ 0& {\mathbf{X}}_{2,BFT}& 0\\ 0& 0& 0\end{array}\right]\left[\begin{array}{l}{\mathbf{p}}_{ADG}\\ {\mathbf{p}}_{BFT}\\ {\mathbf{p}}_{PHS}\end{array}\right]\\ &=\left[\begin{array}{lll}{\mathbf{Z}}_{A,ADG}& 0& 0\\ 0& {\mathbf{Z}}_{A,BFT}& 0\\ 0& 0& {\mathbf{Z}}_{A,PHS}\end{array}\right]\left[\begin{array}{l}{\mathbf{a}}_{ADG}\\ {\mathbf{a}}_{BFT}\\ {\mathbf{a}}_{PHS}\end{array}\right]+\left[\begin{array}{lll}{\mathbf{Z}}_{D,ADG}& 0& 0\\ 0& {\mathbf{Z}}_{D,BFT}& 0\\ 0& 0& {\mathbf{Z}}_{D,PHS}\end{array}\right]\left[\begin{array}{l}{\mathbf{d}}_{ADG}\\ {\mathbf{d}}_{BFT}\\ {\mathbf{d}}_{PHS}\end{array}\right]+\left[\begin{array}{l}{\mathbf{e}}_{ADG}\\ {\mathbf{e}}_{BFT}\\ {\mathbf{e}}_{PHS}\end{array}\right].\end{aligned} $$

Index $j$ is used to denote either ADG, BFT or PHS trait (i.e.,$k=ADG, BFT,PHS$). Term ${\mathbf{y}}_{j}$ is the phenotypic value of individuals, ${{\varvec{\upbeta}}}_{{\varvec{j}}}$ is a vector of systematic effects, ${\mathbf{p}}_{ADG}$ and ${\mathbf{p}}_{BFT}$ are the vector of pen nested within batch effects for ADG and BFT. Terms ${\mathbf{a}}_{j}$ and ${\mathbf{d}}_{j}$ are the vectors of additive and dominance genotypic effects, respectively. Term ${\mathbf{e}}_{j}$ is the vector of residual effects. Matrices ${\mathbf{X}}_{1,j}$, ${\mathbf{X}}_{2,ADG}$**,** ${\mathbf{X}}_{2,BFT}$, ${\mathbf{Z}}_{A,j}$ and ${\mathbf{Z}}_{D,j}$ are incidence matrices that assign the corresponding systematic effects and pen nested within batch effects, additive and dominance genotypic random effects to the phenotypes. There was no correlation between pen nested within batch effects, between residual effects or between these effects and other random effects. The (co)variance matrix for the pen nested within batch effects is $$var\left[\begin{array}{l}{\mathbf{p}}_{ADG}\\ {\mathbf{p}}_{BFT}\\ {\mathbf{p}}_{PHS}\end{array}\right]=\mathbf{I}\otimes \mathbf{P}=\mathbf{I}\otimes \left[ \begin{array}{lll}{\sigma }_{p,ADG}^{2}& 0& 0\\ 0& {\sigma }_{p,BFT}^{2}& 0\\ 0& 0& 0\end{array}\right],$$ where ${\sigma }_{p,ADG}^{2}$ and ${\sigma }_{p,BFT}^{2}$ are the pen nested within batch random variances for ADG and BFT, respectively. The (co)variance matrix for the residuals is $$var\left[\begin{array}{l}{\mathbf{e}}_{ADG}\\ {\mathbf{e}}_{BFT}\\ {\mathbf{e}}_{PHS}\end{array}\right]=\mathbf{I}\otimes \mathbf{R}=\mathbf{I}\otimes \left[\begin{array}{lll}{\sigma }_{e,ADG}^{2}& 0& 0\\ 0& {\sigma }_{e,BFT}^{2}& 0\\ 0& 0& {\sigma }_{e,PHS}^{2}\end{array}\right],$$ where ${\sigma }_{e,j}^{2}$ is the residual variance for each trait. Pen nested within batch effect and the residuals were assumed to be uncorrelated due to overparameterization of the models.

The (co)variance matrix for the additive genotypic effects is

$$var\left[\begin{array}{l}{\mathbf{a}}_{ADG}\\ {\mathbf{a}}_{BFT}\\ {\mathbf{a}}_{PHS}\end{array}\right]={\mathbf{G}}_{0}\otimes \mathbf{G}=\left[\begin{array}{lll}{\sigma }_{A*,ADG}^{2}& {\sigma }_{A*,ADG,BFT}& {\sigma }_{A*,ADG,PHS}\\ {\sigma }_{A*,ADG,BFT}& {\sigma }_{A*,BFT}^{2}& {\sigma }_{A*,BFT,PHS}\\ {\sigma }_{A*ADG,PHS}& {\sigma }_{A*,BFT,PHS}& {\sigma }_{A*,PHS}^{2}\end{array}\right]\otimes \frac{\mathbf{Z}{\mathbf{Z}}^{\mathbf{^{\prime}}}}{\left\{tr([\mathbf{Z}{\mathbf{Z}}^{\mathbf{^{\prime}}}])/n\right\}}$$

whereas the (co)variance matrix for the dominance genotypic effects is

$$var\left[\begin{array}{l}{\mathbf{d}}_{ADG}\\ {\mathbf{d}}_{BFT}\\ {\mathbf{d}}_{PHS}\end{array}\right]={\mathbf{D}}_{0}\otimes \mathbf{D}=\left[\begin{array}{lll}{\sigma }_{D*ADG}^{2}& {\sigma }_{D*ADG,BFT}& {\sigma }_{D*ADG,PHS}\\ {\sigma }_{D*ADG,BFT}& {\sigma }_{D*BFT}^{2}& {\sigma }_{D*BFT,PHS}\\ {\sigma }_{D*ADG,PHS}& {\sigma }_{D*BFT,PHS}& {\sigma }_{D*PHS}^{2}\end{array}\right]\otimes \frac{\mathbf{W}{\mathbf{W}}^{\mathbf{^{\prime}}}}{\left\{tr([\mathbf{Z}{\mathbf{Z}}^{\mathbf{^{\prime}}}])/n\right\}}.$$

Terms ${\sigma }_{A*j}^{2}$ and ${\sigma }_{D*j}^{2}$ are the additive and dominance genotypic variances for each trait, respectively, and ${\sigma }_{A*i,j}$ and ${\sigma }_{D*i,j}$ are the additive and dominance genotypic covariances between the traits.

Terms $\mathbf{Z}$ and $\mathbf{W}$ are incidence matrices relating additive and dominance genotypic effects of either the PB or the CB animals coded as described in the models above, respectively. The prior distributions for the parameters of the model were $P({\varvec{\beta}},b)\sim k$, $P\left({\varvec{p}}\left|\mathbf{P}\right.\right)\sim {\varvec{M}}{\varvec{V}}N(0, \mathbf{I}\otimes \mathbf{P})$, $P(\mathbf{a}\left|{\mathbf{G}}_{0}\right.)\sim {\varvec{M}}{\varvec{V}}N(0, {\mathbf{G}}_{0}\otimes \mathbf{G})$, $P(\mathbf{d}\left|{\mathbf{D}}_{0}\right.)\sim {\varvec{M}}{\varvec{V}}N(0, {\mathbf{D}}_{0}\otimes \mathbf{D})$ and $P\left({\varvec{e}}\left|\mathbf{R}\right.\right)\sim {\varvec{M}}{\varvec{V}}N(0, \mathbf{I}\otimes \mathbf{R})$.

#### Parameter inference

A Bayesian framework was adopted for inference. Flat prior distributions were assumed for the elements of the matrices of the variance components of all the models. Marginal posterior distributions of the parameters of interest were estimated via Gibbs’s sampling algorithm. Mean, highest density interval at 95% (HPD95%) and probability of the parameter of being higher or lesser that certain value, were obtained from these marginal posterior distributions. Univariate models were initially implemented using BGLR software (https://github.com/gdlc/BGLR-R)^21^, and the multiple-trait models were later run with GIBBS2f90 software (http://nce.ads.uga.edu/wiki/doku.php?id=start)^22^. For each proposed model, single chains of 200,000 iterations were run by discarding the first 20,000 iterations. The burn-in was determined by visual inspection of the chains and by the procedures of Raftery and Lewis^23^ and Geweke^24^. Samples of the parameters of interest were saved every 10 rounds.

## Results and discussion

To provide knowledge for determining the best breeding and management strategies in crossbreeding schemes, the magnitude of additive and dominance genotypic correlations between PB and CB performances of three traits of interest in pork production as well as additive and dominance genotypic correlations between these traits within PB and CB populations have been investigated. In addition, the present research has estimated the contributions of genic and non-genic regions to additive and dominance variance for the same traits in pork production in a purebred and a crossbred population.

### Genic and intergenic additive and dominance genetic variances in purebred and crossbred populations

Table 2 shows the additive (i.e., heritability), dominance and pen effect ratios of variance components with respect to the phenotypic variance for ADG, BFT and PHS traits in both PB and CB populations obtained using genotype information of the different genomic regions (i.e., All SNPs, Genic and Intergenic SNPs).

**Table 2:** Mean (SD) of the estimated marginal distribution of heritability (${\mathbf{h}}_{\mathbf{A}}^{2}$), ratio of dominance variance (${\mathbf{h}}_{\mathbf{D}}^{2}$), pen variance (${\mathbf{p}}^{2}$) for average daily gain (ADG), backfat thickness (BFT) and pH semimembranosus (PHS) obtained with univariate genomic models in purebred (PB) and crossbred (CB) populations.

Trait	Population	Model	${{\varvec{h}}}_{{\varvec{A}}}^{2}$	${{\varvec{h}}}_{{\varvec{D}}}^{2}$	${{\varvec{p}}}^{2}$
ADG	PB	All^a^	0.15 (0.08)	0.07 (0.08)	0.20 (0.06)
		Genic^b^	0.15 (0.08)	0.08 (0.08)	0.20 (0.06)
		Intergenic^c^	0.13 (0.08)	0.07 (0.07)	0.20 (0.06)
	CB	All	0.31 (0.11)	0.07 (0.11)	0.17 (0.04)
		Genic	0.27 (0.11)	0.08 (0.12)	0.18 (0.05)
		Intergenic	0.31 (0.10)	0.07 (0.09)	0.17 (0.04)
BFT	PB	All	0.33 (0.11)	0.11 (0.11)	0.09 (0.08)
		Genic	0.33 (0.11)	0.10 (0.10)	0.08 (0.08)
		Intergenic	0.30 (0.11)	0.11 (0.11)	0.09 (0.08)
	CB	All	0.46 (0.11)	0.08 (0.10)	0.10 (0.12)
		Genic	0.44 (0.12)	0.10 (0.06)	0.10 (0.15)
		Intergenic	0.45 (0.10)	0.06 (0.09)	0.10 (0.12)
PHS	PB	All	0.22 (0.08)	0.12 (0.10)	–
		Genic	0.22 (0.08)	0.11 (0.10)	–
		Intergenic	0.20 (0.08)	0.11 (0.10)	–
	CB	All	0.29 (0.09)	0.14 (0.12)	–
		Genic	0.29 (0.09)	0.11 (0.11)	–
		Intergenic	0.28 (0.08)	0.15 (0.10)	–

^a^Univariate genomic model that used all SNPs available to compute additive and dominance genomic relationship matrices.

^b^Univariate genomic model that used SNPs located in genic regions to compute additive and dominance genomic relationship matrices.

^c^Univariate genomic model that SNPs located in intergenic regions to compute additive and dominance genomic relationship matrices.

The results showed that the estimated additive and dominance variance components are relatively the same regardless of the G matrices computed from the various genomic regions (All, Genic, and Intergenic SNPs). This indicates that all genomic regions have nearly the same contribution to the captured genetic variance, either in PB or CB individuals.Our results are in accordance with Do et al.^25^ that indicated that the contribution of each SNP to total genomic variance was similar for genic and non-genic regions on feed intake, daily feed intake, ADG, BFT in pigs. In chickens, Abdollahi-Arpanahi et al.^16^ concluded as well that both genic and non-genic regions contributed to phenotypic variation to body weight, ultrasound measurement of breast muscle and hen house egg production.

Regulatory functions such as promoter, enhancer or transcription factor binding sites are located the upstream and downstream regions of genes, and Gusev et al.^26^ reported that this part of the genome is responsible for genetic variation in 11 human diseases. However, also in humans, Yang et al.^18^ indicated that genic regions described more genetic variation than intergenic regions for height and body mass index. Given the precision of the pig genome annotation and quantity of data available in our study, the hypothesis of different shares of variance for these categories could not be further tested in our dataset. In this study, as a limitation, we used the commercial SNP panel for pigs that was the only one available to us. So, we supposed that the SNPs from genic and non-genic regions from a 60 K commercial SNP chip explain the same amount of genetic variance. However, with availability of the whole genome sequence data it might be a clearer result would be achievable.

Estimated heritabilities were within the range of the previously reported values in Piétrain breed and other pig breeds^27,28^. Heritabilities for the three analyzed traits were higher in CB than in PB populations, however the difference was never significant. This suggests that the gamete effect of the PB population could not be the same when mated within the PB than mated to gamete of another population to produce the crossbreds. Dominance ratio remained of equal magnitude across populations and represented about 7 to 14% of phenotypic variance depending on the trait, suggesting that, for example, assortative matings could slightly enhance both purebred and crossbred performances. In a previous study using the same dataset, different contributions of ratios of dominance deviation variance with respect to the total phenotypic variance were reported across a wide variety of traits related to growth and feed efficiency, carcass composition, meat quality, behavior, boar taint and puberty (ratios ranging from 6 to 18% across 22 traits, Tusell et al.^10^. Our results in some cases were slightly different from the previous study which could be due to the inclusion of the inbreeding effect in the model or related to using the different software’s algorithm^6,29^. Using a model that did not account for inbreeding, the proportion of phenotypic variance explained by dominance was of about 14%, 13% and 8% for lifetime daily gain and 6%, 8% and 10% for backfat weight in Piétrain, Landrace, and Large White populations, respectively^28^. Dominance variance represented about 5% of the phenotypic variance of average daily gain in Duroc pigs using a genotypic model^11^. However, those estimated are genotypic variances, which are slightly different from the variances expressed in terms of breeding values and dominance deviations^13^ of the present study which precludes a proper comparison between estimates.

### Predictive ability

**Figure 1:**
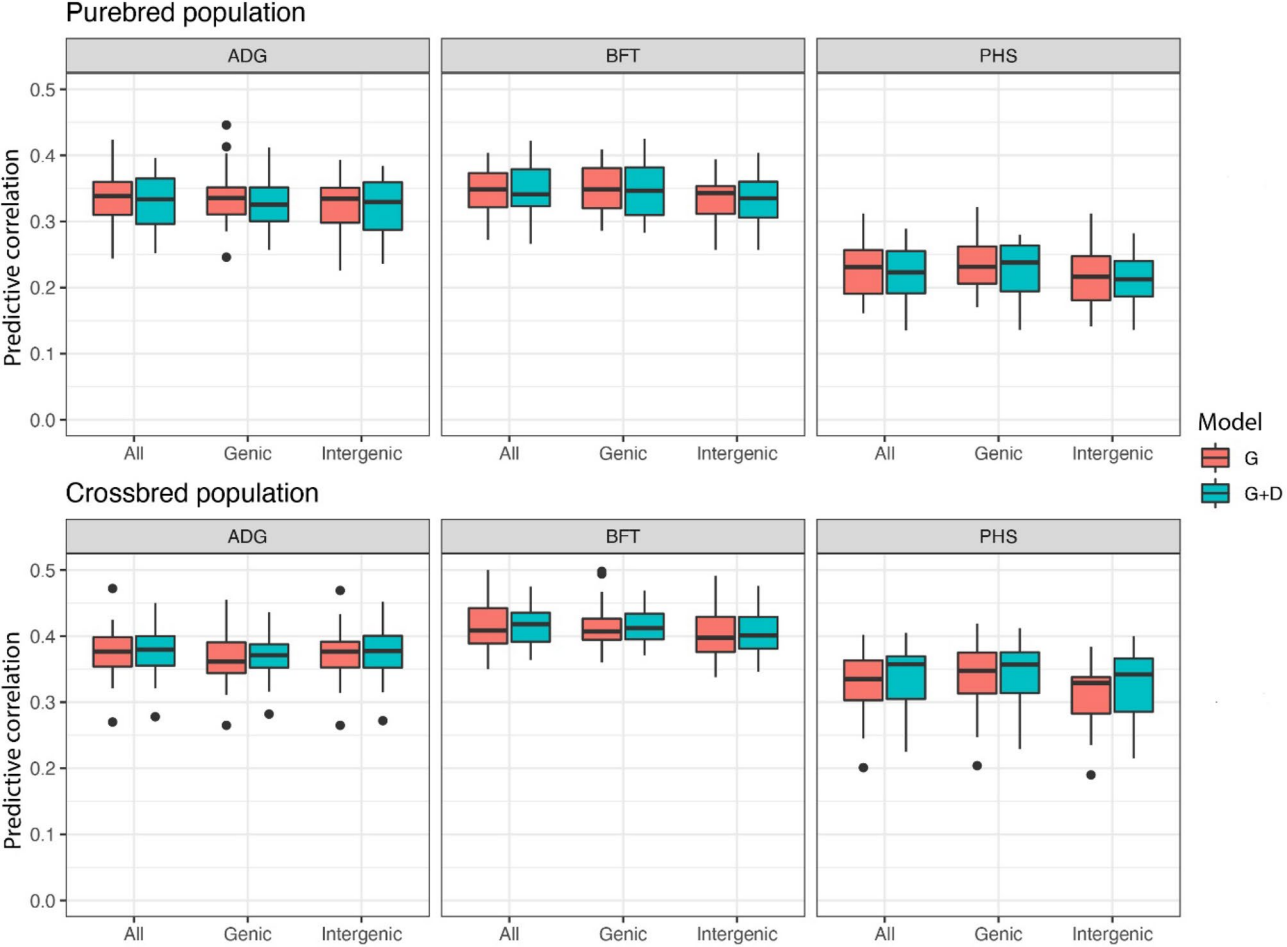
Boxplots of the predictive correlation obtained in the testing sets of fourfold cross-validations repeated ten times of a model with only additive genetic effects (**G**) and a model with additive and dominance genetic effects and inbreeding (**G + D**) using SNPs located in All, Genic and Intergenic regions in purebred and crossbred populations for average daily gain (ADG), backfat thickness (BFT) and pH of the semimembranosus muscle (PHS).

Figure 1 shows boxplots of the predictive correlation obtained in the testing sets of the fourfold cross-validation repeated 10 times of the model with only additive genetic effects and the model with additive plus dominance genetic effects for the three different genomic regions.

All boxplots from the figure overlapped indicating that there were no differences in predictive ability across models within trait and population. Hence, all genomic regions had the same predictive ability. Obtained predictive abilities for ADG and PHS were slightly higher than those obtained in a previous study that used same datasets and slightly different models^30^. This could be because in the previous study, CV folds were split according to the sires to keep all records of the offspring of a sire assigned to the same fold. This precludes an overestimation of the model predictive ability due to strong family relationships between training and testing sets. However, the data split used in the present study does not preclude making fair comparisons between models because both models were evaluated using exactly the same CV split. Our results are in accordance with Do et al.^25^. In that study, predictive accuracy and bias of feed intake, daily feed intake, ADG, BFT in pigs obtained with a model with specific genomic regions did not significantly differ from those models that used randomized SNP groups^25^. Morota et al.^31^, in chickens, found that for body weight and hen house production, non-genic regions performed marginally better than genic regions in terms of prediction ability, whereas for ultrasound area of breast meat, genic regions had a better predictive performance than non-genic regions. However, they indicated that whole-genome distributed SNPs was a better choice for genomic prediction purposes than using only SNPs located in genomic regions.

This was in accordance with Abdollahi Arpanahi et al.^16^ that also found that a whole-genome approach was better for prediction in body weight, ultrasound measurement of breast muscle and hen house egg production in broiler traits than using genomic regions individually. These authors also observed minor differences between classes of genomic regions in terms of predictive ability, being the lowest predictive ability obtained when using missense genomic SNPs. In contrast, Wei et al.^32^ analyzed a natural rice population of 524 accessions with 3,616,597 SNPs to compare the genetic contributions of functionally distinct genomic regions to five agronomic traits. The distributions of the genetic effects revealed that the significant SNPs in non-genic regions generally had larger genetic effects than those in the genic regions. These results support the hypothesis that non-genic regions in the genome are the main source of genetic variations that account for the variability in some complex traits. In our study, we used a smaller number of SNPs (from a standard commercial 60 K SNP chip panel). It would be interesting for further research to investigate in pig populations what would be the contribution to additive and dominance genetic variance of SNPs located in different genomic regions when using a high-density SNP chip panel or whole genome sequence data.

In addition, inclusion of dominance genetic effect and inbreeding in the model did not improve predictive ability already attained with an additive genetic model in the two populations. Xiang et al.^14^ showed that accounting for dominance did not increase the predictive ability of litter size in crossbred pigs but inbreeding depression did.

### Additive and dominance genotypic correlations between PB and CB populations

Table 3 shows estimates of the correlation between purebred and crossbred additive (${rg}_{A}$) and dominance (${rg}_{D}$) genotypic effects obtained in bivariate models where purebred and crossbred performances were considered different traits.

**Table 3:** Mean [highest posterior density interval at 95%] of the correlation between purebred and crossbred additive (${rg}_{A}$) and dominance (${rg}_{D}$) genotypic effects estimated in a bivariate model where purebred and crossbred performances are considered different traits.

Trait	${rg}_{A}$	${rg}_{D}$	P (${rg}_{D}$> 0)^a^
ADG	0.92 [0.74, 1]	0.21 [−0.94, 1]	0.67
BFT	0.83 [0.54, 1]	0.38 [−0.66, 1]	0.77
PHS	0.86 [0.58, 1]	0.30 [−0.67, 1]	0.72

*ADG* average daily gain, *BFT* backfat thickness, *PHS *pH of the semimembranosus muscle.

^a^Probability of a positive dominance genotypic correlation (P (${\mathrm{rg}}_{\mathrm{D}}$> 0)).

Correlation between purebred and crossbred additive genotypic effects were high and positive for the three analyzed traits (${rg}_{A}>0.83$) and none of the highest posterior density interval at 95% included values below 0.50. This indicates that genetic interaction between the Piétrain and Large White breeds for those traits is small in this dataset, which suggests that the genetic progress attained in the purebreds can mostly be completely transferred to the crossbreds as a genetic correlated response. This could be because PB and CB animals of this study were raised in the same environmental conditions, which is not the standard practice in pig breeding schemes where purebred lines are raised in highly sanitized environments and crossbred animals in commercial farms. Using the same dataset in a previous study^33^, genetic correlation between purebred and crossbred was estimated to be of 0.50 for ADG and 0.94 for PHS. Dugué et al. (2020), used bivariate models but did not account for dominance genetic effects. We believed that incorporating dominance effects and inbreeding coefficient simultaneously can improve phenotypic prediction accuracy. Wientjes and Calus^5^ reviewed 201 purebred-crossbred correlation estimates reported from 27 studies in pigs and the average estimate was 0.63, with 50% of the estimates between 0.45 and 0.87. These values indicate that for those correlations departing from unity, accounting for crossbred performance in genetic evaluations of purebred candidates could be advisable for boosting crossbred performance. In a further step, Xiang et al.^14^ estimated for the first time the correlations of allele substitution effects between purebred and crossbred performance for Landrace and Yorkshire breeds and proposed these correlations as consistency measurements of SNP substitution effects across breeds. For purebred performance, it was estimated to be 0.19 with a high imprecision indicating that SNP effects estimated in one breed cannot be readily applied to the other breed. For crossbred performances, this figure was estimated to be 0.98 indicating that SNP effects estimated in a crossbred population can be used to estimate crossbred breeding values in the two purebred parental populations^14^.

Estimates of the correlation between purebred and crossbred dominance genotypic effects were highly imprecise, precluding us to have strong evidence of the magnitude and sign of this parameter across the analyzed traits. However, the probability of a positive value was 0.67, 0.72 and 0.77 for ADG, PHS and BFT, respectively. The estimates of this parameter reported in the literature are scarce and imprecise. Dominance genotypic correlations for litter size between two pure lines and a crossbred pig population were estimated to be 0.47 ± 0.41 and 0.59 ± 0.36^12^. Further research with more data would improve the precision of these estimates and may allow being conclusive about if underlying genetic mechanisms responsible for the dominance effects differ between PB and CB populations. If this were the case, mate allocation strategies to improve total genetic value of the descendants would differ between populations.

Ratios of additive genetic variance (i.e., heritability), dominance genetic variance and pen nested within batch variance with respect to the phenotypic variance estimated via the bivariate models of PB and CB performances for the three traits are shown in supplementary file S1. All of them were consistent and within the range of the estimates obtained with the univariate models using all SNPs discussed above so they are not discussed here.

### Additive and dominance genotypic correlations between BFT, ADG and PHS in PB and CB populations

Table 4 shows the estimates of the additive and dominance genotypic correlations between ADG, BFT and PHS obtained separately in the purebred and the crossbred population.

**Table 4:** Mean [highest posterior density interval at 95%] of the estimated marginal distribution of the additive and dominance genotypic correlations between traits estimated in a purebred (PB) and a crossbred (CB) population.

Traits		${rg}_{A}$^a^	${rg}_{D}$^b^	P (${rg}_{D}$> 0)^c^
ADG, BFT	PB	0.83 [0.31, 1]	0.11 [−0.61, 0.86]	0.62
	CB	0.30 [−0.11, 0.65]	0.77 [0.47, 0.99]	0.99
ADG, PHS	PB	−0.70 [−1, −0.24]	−0.34 [−0.99, 0.56]	0.26
	CB	0.15 [−0.39, 0.69]	−0.15 [−0.57, 0.30]	0.23
BFT, PHS	PB	−0.57 [−0.97, −0.22]	0.55 [0.14, 0.95]	0.98
	CB	−0.66 [−0.97, −0.14]	0.46 [0.09, 0.87]	0.98

*ADG* average daily gain, *BFT *backfat thickness, *PHS* pH of the semimembranosus muscle.

^a^Additive genotypic correlation (${\mathrm{rg}}_{\mathrm{A}}$).

^b^Dominance genotypic correlation (${\mathrm{rg}}_{\mathrm{D}})$.

^c^Probability of a positive dominance genotypic correlation (P (${\mathrm{rg}}_{\mathrm{D}}$> 0)).

Additive genetic correlation between ADG and BFT was estimated to be high in PB and moderate in CB, being the latter more in the range of previous reported estimates^34–37^ . Additive genetic correlation between ADG and PHS was estimated to be highly negative and moderately positive in PB and CB populations, respectively, the latter being in accordance with the estimate obtained by Lo et al. in Duroc pigs (0.24 ± 0.11)^38^. Although estimates are highly imprecise, this is a contradictory result, as one would expect to encounter an estimate given that genetic correlations for ADG and pH between PB and CB populations were high. A clearly negative genetic correlation between BFT and PHS was estimated in PB and CB populations used in the present study, whereas it was estimated moderate and positive in Duroc pigs (0.47 ± 0.10)^38^. Genetic relationships between growth performance and meat quality traits are scarce and not clear in pigs^35^ but the reported estimates suggest that genetic correlations are highly influenced by the breed type.

Estimates of dominance genotypic correlations were very imprecise between all pair of traits in both populations. This is not surprising since it is more difficult to estimate accurately covariance between effects if the variances of these effects are low. However, there is some evidence of positive value for some of the estimated dominance genotypic correlations because the probability of a positive dominance genotypic correlation was high between ADG-BFT and BFT-PHS. Additive and dominance genotypic correlations between BFT and PHS were of different sign. This makes it difficult to discuss its implications, but one can hypothesize that genes contributing to the additive genetic progress in both traits would have an antagonistic effect when used for exploiting dominance effects in planned matings. to our knowledge, there are no reported estimates of dominance correlations in the literature for these traits, which precludes us from making further discussion about these estimates. Nonetheless, the different values of dominance genotypic correlations between traits indicate that maximizing total genetic gain of several traits simultaneously might not be straightforward.

## Conclusions

In this study, genic and intergenic regions were able to capture the same genomic relationships among either PB or CB populations. Correlations between purebred and crossbred additive genotypic effects were high and positive whereas estimates of the correlations between purebred and crossbred dominance genotypic effects were highly imprecise for the three traits analyzed. The later precludes us being conclusive about if genetic mechanisms responsible for the dominance effects differ between PB and CB populations. Estimates of dominance genotypic correlations were imprecise but with strong evidence of positive values for ADG-BFT and BFT-PHS. Additive and dominance genotypic correlations between BFT and PHS were of different sign in both populations indicating that genes contributing to the additive genetic progress in both traits would have an antagonistic effect when used for exploiting dominance effects in planned matings. The different values of dominance genotypic correlations between traits indicate that maximizing total genetic gain of several traits simultaneously would not be straightforward. Finally, all genomic regions lead to the same predictive ability, that did not improve with the inclusion of dominance genetic effects and inbreeding in the models.

## Supplementary Information

Supplementary Table S1.

## Data Availability

The data that support the findings of this study are co-own by INRAE, IFIP and Alliance R&D association (Axiom, Choice Genetics France, Nucléus, IFIP). Restrictions apply to the availability of these data, which were used under license for this study.
