# Supplementary material for: Genic and non-genic SNP contributions to additive and dominance genetic effects in purebred and crossbred pig traits
Source: Sci Rep. 2022 Mar 9;12:3795. doi: 10.1038/s41598-022-07767-3 (PMC8907311; doi:10.1038/s41598-022-07767-3)
Supplement: Supplementary file 1 — Supplementary Table S1. [file 41598_2022_7767_MOESM1_ESM.docx]

Supplementary table 1. Mean [highest posterior density interval at 95%] of the estimated marginal distribution of the ratios of additive genetic variance ($h_{A,k}^{2}$, i.e. heritability), dominance genetic variance ($h_{D,k}^{2}$), pen nested within batch variance ($p_{k}^{2}$) with respect to the phenotypic variance ${(\sigma}_{\boldsymbol{k}}^{\boldsymbol{2}}\boldsymbol{)}$ estimated in a bivariate model where, purebred (*k*=PB) and crossbred (*k*=CB) performances considered different traits in bivariate models.

| Parameter | **ADG^1^** | **BFT^2^** | **PHS^3^** |
| --- | --- | --- | --- |
| $\boldsymbol{h}_{\boldsymbol{A,PB}}^{\boldsymbol{2}}$ | 0.19  [0.11, 0.28] | 0.29  [0.18, 0.38] | 0.20  [0.12, 0.28] |
| $\boldsymbol{h}_{\boldsymbol{A,CB}}^{\boldsymbol{2}}$ | 0.28  [0.21, 0.35] | 0.35  [0.28, 0.42] | 0.25  [0.19, 0.33] |
| $\boldsymbol{h}_{\boldsymbol{D,PB}}^{\boldsymbol{2}}$ | 0.04  [0.00, 0.11] | 0.08  [0.00, 0.17] | 0.07  [0.00, 0.15] |
| $\boldsymbol{h}_{\boldsymbol{D,CB}}^{\boldsymbol{2}}$ | 0.07  [0.01, 0.13] | 0.08  [0.09, 0.14] | 0.10  [0.03, 0.16] |
| $\boldsymbol{p}_{\boldsymbol{PB}}^{\boldsymbol{2}}$ | 0.15  [0.07, 0.23] | 0.03  [0.00, 0.06] | - |
| $\boldsymbol{p}_{\boldsymbol{CB}}^{\boldsymbol{2}}$ | 0.12  [0.06, 0.19] | 0.06  [0.02, 0.10] | - |
| $\sigma_{\boldsymbol{PB}}^{\boldsymbol{2}}$ | 10919  [9097, 12872] | 4.24  [3.44, 5.07] | 0.04  [0.03, 0.05] |
| $\sigma_{\boldsymbol{CB}}^{\boldsymbol{2}}$ | 11646  [9859, 13434] | 6.41  [5.40, 7.46] | 0.04  [0.04, 0.05] |

^1^average daily gain (ADG), ^2^backfat thickness (BFT), ^3^pH of the semimembranosus muscle (PHS).
